# Extraordinary Case of Diseased Uterus

**Published:** 1815-08

**Authors:** Thomas Sewall


### *Extraordinary Case of diseased Uterus;* by THOMAS SEWALL, M.D.

IN the spring of 1808, I was called to attend the dissection of the extraordinary case of diseased uterus in Miss M. a young woman aged about twenty. She was below the middling stature, rather slender, but had generally enjoyed a tolerable state of health. The history of her singular case is as follows. About eighteen months before her decease, she experienced a sudden cessation of the menses, and acknowledged herself guilty of an act of illicit connexion with the other sex ; from this she supposed conception had taken place, and accordingly made an avouch before a justice of the peace. From the first ceasing of the menses there was a gradual and uniform enlargement of the abdomen and breasts, and the first eight months were marked by most of the circumstances usual in an ordinary case of gestation. At the end of nine months she supposed herself ill, called in a physician, who, on examination, discovered no symptoms of labour, and left her. From this time the enlargement of the abdomen was more rapid and the breasts became hard and inelastic. At the end of nine months more she expired, apparently from the great distention of the abdomen and extreme emaciation.

Two days after death the body was inspected in presence of a large number of medical gentlemen.

The superficial veins of the abdomen had become varicose to a considerable extent. On laying open the abdomen the uterus was found occupying nearly its whole cavity, with its surface irregular and adhering in many points to the peritoneum, neum. This, when dissected out, weighed thirty-two pounds and an half. On examining its internal structure, there were found two cavities, one the natural cavity of the uterus, whose surface was tolerably smooth and regular, containing a dark glumous fluid to the amount of a number of pounds, the other senum and pus. These were evacuated, and the uterus weighed twenty-one pounds. The os tincæ had adhered, and the ovaria were enveloped in the diseased mass.

In cutting into the substance of the uterus it exhibited generally a whitish color, a very firm unyielding texture intersected by membranous septa. Some points were cartilaginous and others approaching to the state of bone. The intestines were crouded back on the spine, and some of the mesenteric glands enlarged to the size of grapes and in a state of induration. The other abdominal viscera were in a healthy state.

It may be regarded as a remarkable fact, that, during the whole course of this disease, the appetite was unusually voracious, craving constantly animal food, which was taken and perfectly digested, the bowels were most of the time firm and regular. THOMAS SEWALL.

JOHN C. WARREN, M.D. *Boston.*

---

